# Novel Robotic Balloon-Based Device for Wrist-Extension Therapy of Hemiparesis Stroke Patients

**DOI:** 10.3390/s25051360

**Published:** 2025-02-23

**Authors:** Klaudia Marek, Aleksandra Olejniczak, Elżbieta Miller, Igor Zubrycki

**Affiliations:** 1 Department of Neurological Rehabilitation, Medical University of Lodz, Milionowa 14, 93-113 Lodz, Polandelzbieta.dorota.miller@umed.lodz.pl (E.M.); 2 Institute of Automatic Control, Lodz University of Technology, Stefanowskiego 18, 90-537 Lodz, Poland; igor.zubrycki@p.lodz.pl

**Keywords:** rehabilitation robot, wrist, upper limb, stroke, hemiparesis

## Abstract

Upper-limb paresis is one of the main complications after stroke. It is commonly associated with impaired wrist-extension function. Upper-limb paresis can place a tremendous burden on stroke survivors and their families. A novel soft-actuator device, the Balonikotron, was designed to assist in rehabilitation by utilizing a balloon mechanism to facilitate wrist-extension exercises. This pilot study aimed to observe the functional changes in the paralyzed upper limb and improvements in independent and cognitive functions following a 4-week regimen using the device, which incorporates a multimedia tablet application providing audiovisual feedback. The device features a cardboard construction with a hinge at wrist level and rails that guide hand movement as the balloon inflates, controlled by a microcontroller and a tablet-based application. It operates on the principle of moving the hand at the wrist by pushing the palm upwards through a surface actuated by a balloon. A model was developed to describe the relationship between the force exerted on the hand, the angle on hinge, the pressure within the balloon, and its volume. Experimental validation demonstrated a Pearson correlation of 0.936 between the model’s force predictions and measured forces, supporting its potential for real-time safety monitoring by automatically shutting down when force thresholds are exceeded. A pilot study was conducted with 12 post-stroke patients (six experimental, six control), who participated in a four-week wrist-extension training program. Clinical outcomes were assessed using the Fugl–Meyer Assessment for the Upper Extremity (FMA-UE), Modified Rankin Scale (mRS), Mini-Mental State Examination (MMSE), Montreal Cognitive Assessment (MOCA), wrist Range of Motion (ROM), and Barthel Index (BI). Statistically significant results were obtained for the Barthel index (*p* < 0.05) and FMA-UE, indicating that the experimental use of the device significantly improved functional independence and self-care abilities. The results of our pilot study suggest that the Balonikotron device, which uses the principles of mirror therapy, may serve as a valuable adjunct to conventional rehabilitation for post-stroke patients with hemiparetic hands (BI *p* = 0.009, MMSE *p* = 0.151, mRS *p* = 0.640, FMA-UE *p* = 0.045, MOCA *p* = 0.187, ROM *p* = 0.109).

## 1. Introduction

Many post-stroke patients in the subacute phase have problems with wrist extension and flexion and finger extension [1,2]. Hand paralysis continues to be a major rehabilitation challenge [3], up to 60 percent of post-stroke patients are reported to have upper-limb motor impairment [4]. Reduced sensorimotor control is one of the most common effects of stroke [5]. Upper-limb limitations reduce quality of life in stroke survivors. They result in functional, cognitive, social, physical or emotional problems [6,7]. Upper-limb paresis can pose a huge burden on post-stroke individuals and their families. Restoration of upper-extremity function remains a major challenge for medical teams and physiotherapists due to the lack of effective treatment strategies [8]. Impaired wrist function is particularly problematic. Wrist extension is an essential part of the movement of the hand in grasping an object. Without this ability, many motor tasks cannot be performed [3]. According to Compos et al. (2019), even several years after stroke, patients in the chronic phase showed significant limitations in activity and participation. Limitations included use of communication devices, home life, social and civic life, and application of knowledge [9].

Hemiplegia is defined as complete or partial weakness of one side of the body. Weakness occurs in about 80–90 percent of stroke patients and is a major disability affecting daily life [10,11]. Acute limb paresis can lead to complication of long-term rehabilitation and recovery of motor function [12]. Motor learning is an essential component of neurological rehabilitation. Regeneration and brain plasticity are stimulated by specific exercises or intensive, high-repetition training [13,14].

Previous studies have suggested that training the wrist joint, particularly the wrist extension, can improve the hand function [3,15,16,17,18]. Li et al. (2021) used an electroencephalogram (EEG) to construct the relevant time-varying networks critical for wrist extension. Effective connectivity between the stroke-affected motor area and other areas was reduced. Increased connectivity between motor areas and other areas, particularly between the frontal and parietal–occipital lobes, has been found during wrist-extension performance in post-stroke patients, suggesting dynamic compensation for motor and behavioral deficits [3]. Cross-training, which plays a role in the case of unilateral motor deficits, is also applicable here. This term refers to the training of one side of the body to increase the strength of analogous muscles on the untrained side. Cross-education for neurological diseases, such as paresis, involves focusing on training the unaffected side [19].

Studies suggest that the use of upper-extremity mirror therapy in post-stroke patients results in improvements in motor function, activities of daily living, and pain reduction [20,21,22]. Mirror therapy was originally used for amputees with phantom limb pain [23]. The therapy is described as a form of motor imagery and action observation technique using the concept of visual illusion. Used as a stand-alone therapy or in combination with other methods, it provides feedback to the patient through visual illusion [24,25,26]. A mirror is placed between the healthy and active limb and the diseased and inactive limb. The neurological patient receives a reflected illusion of the damaged limb moving in front of the mirror. During MT, the patient observes in a mirror the reflection of the healthy hand that performs the movement. This leads to the regeneration of nerve connections in the damaged area of the brain after cerebrovascular disease [27]. The advantage of mirror therapy is that it can be used for severe, even complete, limb paralysis in stroke survivors. This is possible because the therapy uses visual stimuli to influence the induction of the desired response in the affected limb [28].

The design of robots with the idea of mirror therapy for rehabilitation has a longer record. Pu et al. (2020) designed a robot for stroke patients for hand rehabilitation with elements of mirror therapy. The robotic system allowed passive movement exercises as well as mirror-therapy pinching and grasping movements under glove control. The robot is actually a hand therapy support system designed for stroke patients. Two exercise modes allow mirror therapy training and passive range of motion of the hand joints. The unaffected hand performs movements that are recorded by sensors in the glove. The exoskeleton mimics the recorded movements on the patient’s disabled hand [29]. Robots have been used successfully in rehabilitation for many years [30,31]. The shortage of physiotherapists influences the development of new robotic ideas [32]. An important feature in robotic rehabilitation is a system that helps the patient achieve and perform limited movement, rather than passively moving the paralyzed limb [33].

Cognitive dysfunctions, including executive, memory, perceptual and emotional impairments, are increasingly common in post-stroke patients. Cognitive dysfunction includes executive and working memory, visuospatial, and emotional deficits associated with depressive/anxiety problems [34,35]. A recent study by Lee et al. suggests that mirror therapy is effective in post-stroke patients with cognitive dysfunction and has a positive effect on rehabilitation [36].

There are many hand-rehabilitation devices available in the medical industry. The field of upper-limb robotics in neurological rehabilitation is developing rapidly with the emergence of new commercial devices [37]. SaeboFlex is a dynamic orthosis for the wrist, hand, and fingers. It is designed to activate patients in daily activities when there is difficulty with decreased wrist and finger function and upright movement [38]. Barriers cited by clinicians observing patients using the orthosis include the need for assistance in putting on and taking off the orthosis, and the high cost, which may be out of patients’ financial reach [39]. Several robotic solutions use virtual games with visual feedback in training [40]. The Exo-Wrist robot developed by Choi et al. (2019) assists wrist movement using actuators. It is a soft device that uses a tendon actuator to generate force to assist wrist movement. The movement performed is oblique, a combination of upright, flexion, abduction, and adduction. The splint, which forms the base of the robot, is printed in a 3D printer and customized for the patient based on wrist measurements [41].

Soft robotic devices for hand rehabilitation offer significant advancements over those designed with rigid links and actuators. These devices are made of non-rigid materials, providing inherent compliance and reducing potential joint damage. They can also be lighter and potentially simpler. Chu and Patterson, in a narrative review paper of soft robotic devices for hand rehabilitation, note that the field is advancing rapidly, with 44 such devices identified in their study [42]. In addition to the many advantages of soft robots, such as the mobility and lightness of the device, the individual and precise fit to the limb, and the possibility of using the robot with other assistive devices, it is also important to consider the disadvantages. Soft robots have potential disadvantages: prolonged adherence to the body, which can cause compression and the development of a blood flow problem; the possibility of deformation of the soft structures of the robot; skin abrasions; and difficulty in maintaining the path of the cable; they may have difficulties in anchoring to the patient’s body [41]. However, the authors mention that there is room for improvement, particularly in the areas of patient feedback, user intent detection, and safety, especially with respect to joint motion limits and device portability. In our paper, we describe a new minimalist, soft actuator-based hand extension device that focuses on human interaction through mirror therapy. It is battery-powered and portable, and incorporates safety features based on joint limits and calculated force applied to the hand. The aim of this case study was to determine the functional changes in upper-limb recovery, independence, cognitive function, and muscle tone induced by 4 weeks of rehabilitation using the device with audiovisual application with elements of mirror therapy. We are verifying our hypothesis that a minimalist Balonikotron device with an audiovisual cat application, designed for controlled wrist-extension training, has a potentially beneficial use in post-stroke neurological rehabilitation. It was hypothesized that rehabilitation with the device would improve hemiparetic hand function, cognitive function, and patient independence.

## 2. Materials and Methods

### 2.1. Balonikotron Device

**Figure 1:**
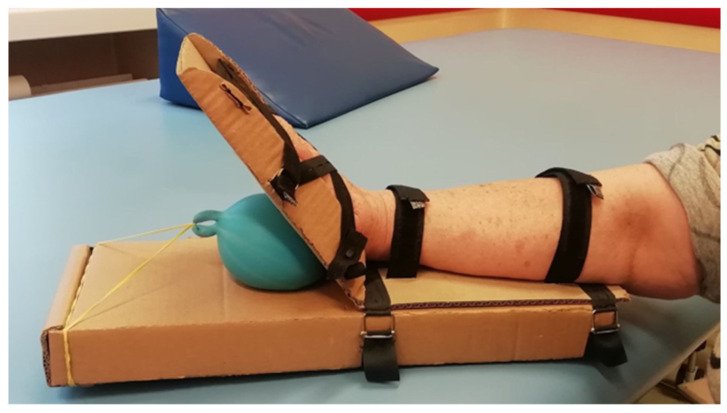
The device during extension exercise: balloon inflation pushes the upper, hinged component on which the hand is placed. The hand extends and slides in the rail.

The Balonikotron device is designed as a single-patient use, recyclable, and portable system to support hand rehabilitation by facilitating wrist-extension exercises. It operates using a balloon-actuated mechanism that lifts the palm, enabling controlled wrist extension. The device features a lightweight cardboard structure with a hinge at the wrist level and guiding rails to assist hand movement during inflation. The balloon, securely fastened within the base, has its neck threaded through a hole in the base and secured in place, preventing movement during inflation. As the balloon expands between two surfaces, it induces the desired extension motion (Figure 1).

The device’s construction prioritizes hygiene and patient safety, with the main cardboard surface intended for single-patient use. This component can be detached and replaced using a belt-based attachment system, ensuring continued usability while maintaining sterility. The disposable base allows for easy replacement in case of contamination or damage. Built-in straps provide optional limb stabilization but are not mandatory, allowing flexibility based on patient needs. In cases where tightening could cause discomfort, increased muscle spasms, or sensory disturbances associated with limb paresis, the straps can remain loose.

The device has a diaphragm pneumatic pump with its driver, a valve allowing air to escape to the atmosphere, and an angle sensor based on a resistive flex sensor. The device is controlled by an ESP32-based board with an additional screen and capacitive switches attached. It is battery-operated with a LiPo battery and a battery management system housed inside the device. The mirror-therapy-inspired interface used in this study is described in the chapter below.

The main component of the device is the balloon, located between two walls, one of which has a hinge mounted very close to it. As the balloon inflates, it expands between the walls and presses against them. The balloon exhibits a non-linear expansion characteristic, where beyond a certain pressure, further air pumping increases the volume but does not significantly increase the pressure.

It is beneficial if the device of this type has a safety feature based on applied force to a hand. However, directedly measuring force would require adding additional sensing circuitry to the externals of the device. Instead of the amount of air inside the balloon, the angle of the hinged component and the pressure can be used to estimate the force. While the exact model of the balloon behavior would be hard to run on the device controller, a simplified model can be developed for online estimation of the force applied. While running a precise model of balloon behavior on the device controller would be computationally demanding, a simplified model can be developed for real-time force estimation.

**Figure 2:**
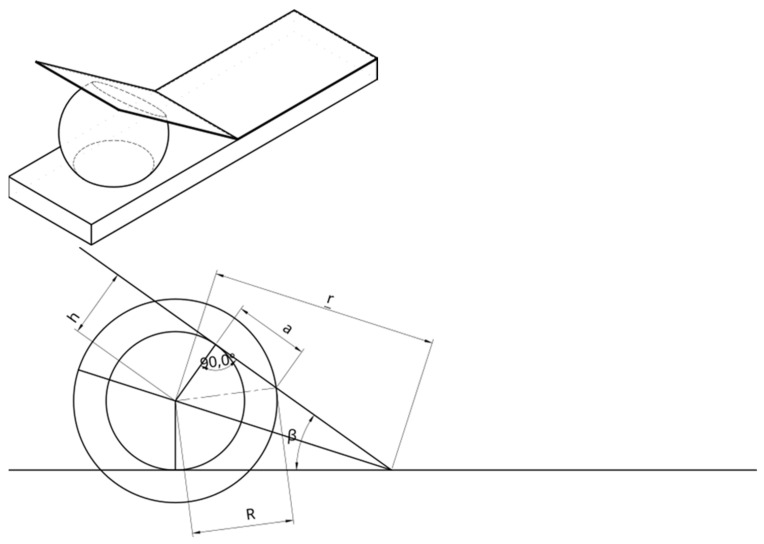
Presentation of a basic concept of a Balonikotron device.

A relationship of force in relation to air pressure, air volume, and the angle between surfaces can be developed by modeling the balloon as a segment of a sphere truncated by two planes (the device’s walls). The volume can be then expressed as a volume of a sphere minus the volume of two spherical cups. The center of the sphere is placed at a distance r from the edge; we assume that it is centered between two walls as shown in Figure 2. Further, we assume that distance does not change during operation, as the balloon’s neck is attached to the base through the hole placed close to the edge.

Using this model, the volume of air trapped by the balloon will depend on the radius of the ball R, the angle beta, and the distance of the center of the balloon from the edge r:

$$h=r\sin\left(\frac{\beta }{2}\right)$$

$$a=\sqrt{R^{2}-h^{2}}$$

Therefore, the volume of single spherical cup is

$$V_{c}=\frac{\pi {\left(R-r\sin\left(\frac{\beta }{2}\right)\right)}^{2}\left(2R+r\sin\left(\frac{\beta }{2}\right)\right)}{3}=\frac{2\pi R^{3}}{3}-\pi R^{2}r\sin\left(\frac{\beta }{2}\right)+\frac{\pi r^{3}\sin^{3}\left(\frac{\beta }{2}\right)}{3}$$

and the volume of ball without the cups is

$$V_{b}=2\pi R^{2}r\sin\left(\frac{\beta }{2}\right)-\frac{2\pi r^{3}\sin^{3}\left(\frac{\beta }{2}\right)}{3}$$

From this equation, we can determine how the contact area S of the balloon pressing on the platform (a circle with radius α) depends on the volume of air in the balloon and the angle β.

$$R^{2}=\frac{V_{b}+\frac{2}{3}\pi r^{3}\sin^{3}\left(\frac{\beta }{2}\right)}{2\pi r\sin\left(\frac{\beta }{2}\right)}$$

$$S=\pi a^{2}=\pi \left(R^{2}-r^{2}\sin^{2}\left(\frac{\beta }{2}\right)\right)=\frac{V_{b}-\frac{4}{3}\pi r^{3}\sin^{3}\left(\frac{\beta }{2}\right)}{2r\sin\left(\frac{\beta }{2}\right)}$$

Force applied to the hand through the platform is related to pressure inside the balloon and the surface of the balloon being in contact with the platform. The contact the force depends on the internal pressure and the contact area S:

$$F(\beta ,V_{b},\rho )=\rho \;S=\rho \;\frac{V_{b}-\frac{4}{3}\pi r^{3}\sin^{3}\left(\frac{\beta }{2}\right)}{2r\sin\left(\frac{\beta }{2}\right)}$$

where β is angle of the platform, $V_{b}$ is volume of air in the balloon, ρ is air pressure inside the balloon, and *r* is the distance from the center of the balloon to the edge (hinge).

To validate the force estimation model, a test setup was used to measure force, angle of the plate, air flow, and pressure for angles ranging from 9 to 35 degrees. The recorded trajectories were then compared to the model predictions, yielding a Pearson correlation coefficient of 0.936, indicating a strong agreement. The characteristic radius parameter (r) was determined using SciPy’s optimization function, which minimized the least-squares error between the modeled and measured forces, resulting in an estimated value of 16 cm.

The model can be further simplified (to remove the need for particular sensors) observing that the pressure in the balloon does not change linearly while expanding but is nearly constant for large changes in volume. Also, the diaphragm pump used in the device pumps has a nearly constant (average) flow during its operation, and the flow of air back to the atmosphere from the balloon is also nearly constant (as it depends on air resistance and near-constant pressure inside the balloon). This allows for a simplified model where force on the platform can be estimated using only the angle of the platform and duration of pump operation.

$$V_{best}=t_{on}\cdot k_{in}-t_{off}\cdot k_{out}$$

where *V_best_* is an estimated volume of the air in the balloon, *t_on_* is the duration that the pump was on, *k_in_* is the constant air flow generated by the pump, *t_off_* is the duration that the the pump is off, and *k_out_* is the constant outflow to the atmosphere.

$$F_{simp}\left(\beta ,t_{on},t_{off}\right)=\rho \;S=\rho_{const}\frac{V_{best}\left(t_{on},t_{off}\right)-\frac{4}{3}\pi r^{3}\sin^{3}\left(\frac{\beta }{2}\right)}{2r\sin\left(\frac{\beta }{2}\right)}$$

**Figure 3:**
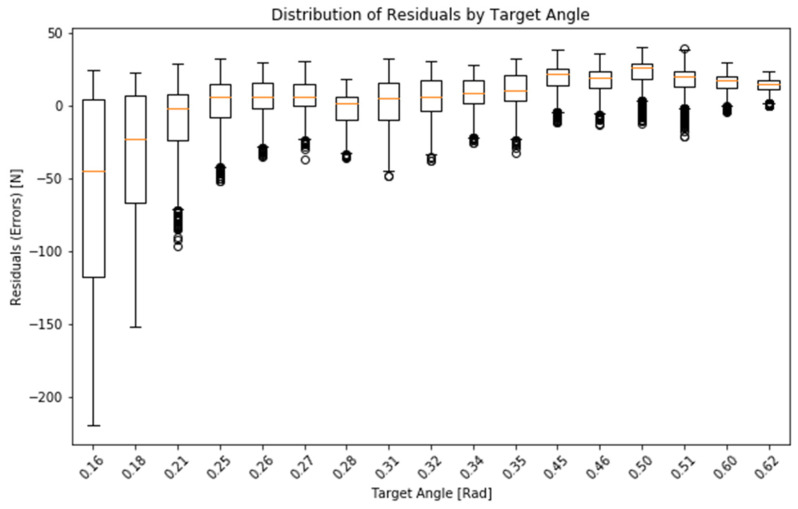
Distribution of residuals by target angle.

**Figure 4:**
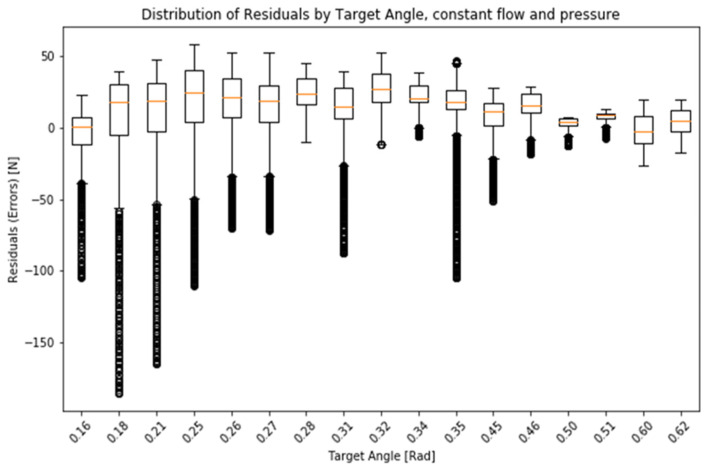
Distribution of residuals by target, angle, constant flow, and pressure.

Such a simplified model has a standard (Pearson) correlation between the model and the recorded force of 0.930 in the experimental setting. See Figure 3 and Figure 4.

**Figure 5:**
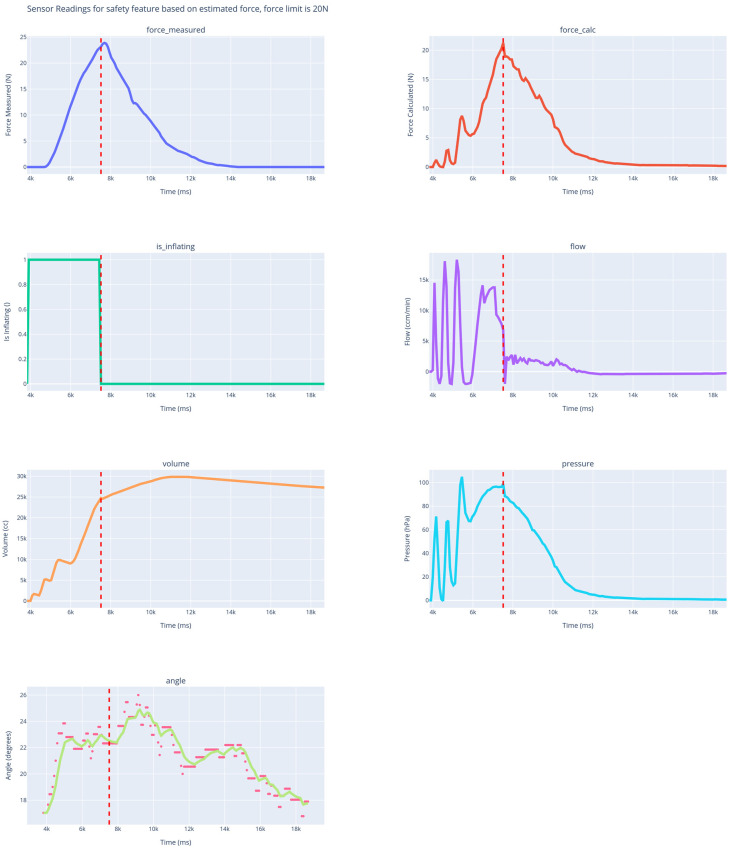
Sensor readings from the implementation of the model-based safety feature. The balloon deflation triggers (red line) when the model estimates 20 N of force.

Figure 5 shows the sensor readings for the device with force estimation based on the pressure sensor (MPRS pressure sensor by Adafruit, Adafruit - USA/New York), flow sensor (SFM4100 by Sensirion—note that for small inverted flow (balloon deflation), the sensor readings did not register correctly), and flex sensor (digital flex sensor by Bend Labs). The readings indicate that the estimation accurately tracks changes in the actual force. The safety feature, programmed for 20 N, triggered balloon deflation at 23 N, with a maximum applied force of 23.8 N. The sampling/control period was 85 ms, and the maximum angular velocity was 7 degrees per second.

### 2.2. Function and Interface of the Balonikotron Device

The Balonikotron device is designed for the rehabilitation of the hemiparetic hand. While the device can be used in a fully automatic mode (repeating a time-based pattern), in the study, a mirror-therapy-inspired interface was used. In this mode, the device and its interface consist of three parts: a follower device, which moves the hand as described above; a leader device, which is an additional board attached to the patient’s healthy hand; and a tablet with an interface for patient training. The tablet application guides the user during the therapy session (and breaks) and displays a cat avatar that gives the patient voice commands to extend the wrist and return to the initial position (raising the hand up and lowering it back down).

**Figure 6:**
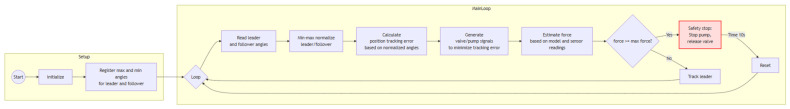
Control flow in tracking/mirror mode of the Balonikotron device, with safety mode based on estimation of force. The minimalization of tracking error in the version of the device used in the experiments described in later chapters was based on a bang-bang controller with hysteresis, and the safety check was not used.

The scheme of leader–follower interaction is very similar to classical teleoperation where the follower device with patient’s affected hand moves in accordance to the patient’s healthy hand movement in the leader device. However, for patient safety, the movement is mapped according to the recorded range of motion of both the healthy and affected hands; min–max normalization is used. Both extension angle values are normalized, and the device tracks the (normalized) leader value using a regulator for reducing the tracking error (a bang-bang controller with hysteresis was used in the experiments below). The logic of the setup with the additional safety feature based on estimated force is shown in Figure 6.

**Figure 7:**
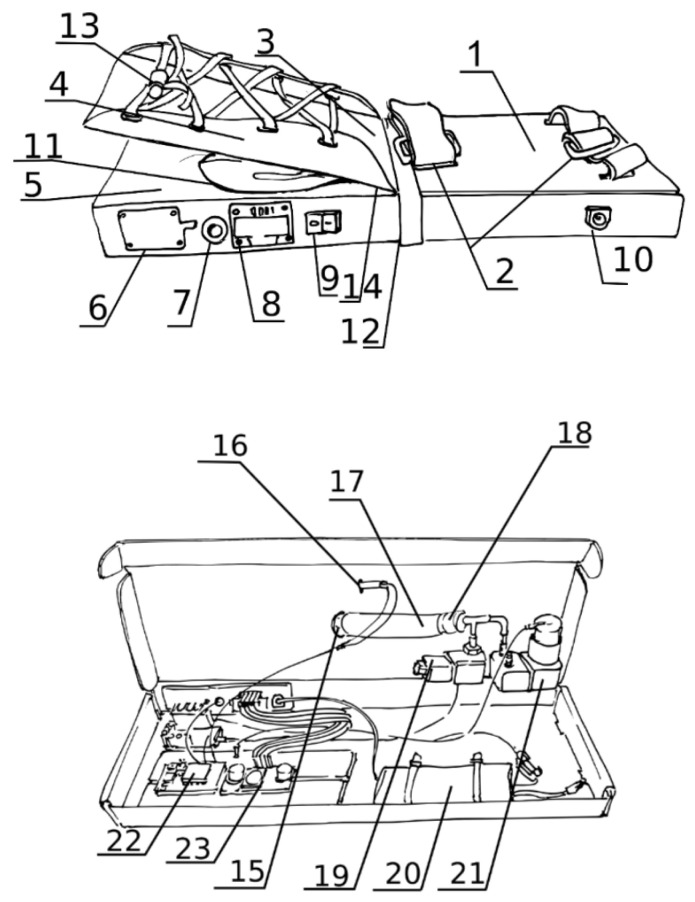
Details of construction. 1. Main board, 2. Forearm stabilization system, 3. Movable stabilizing plate, 4. Sliding insert, 5. Device housing, 6. Control button, 7. LED indicator, 8. Display screen, 9. Power button, 10. External power supply port, 11. Balloon (Membrane actuator), 12. Hinge, 13. Hand stabilization system, 14. Flexion sensor, 15. 16: Flex sensor cables 17: Pneumatic tube 18: Pneumatic T-connector 19: Membrane pump 20: Battery 21: Electro-pneumatic valve 22: Control board 23: Battery control circuit (now integrated into the control board). Hole to insert the neck of the balloon. Figure taken from the patent application of Zubrycki and Koter with permission [43].

A more detailed picture of the construction of the device (inside and outside) is shown in the following Figure 7.

**Figure 8:**
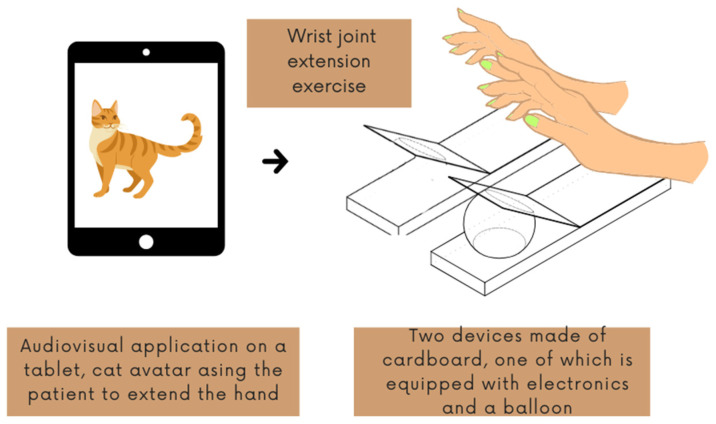
Diagram of the interaction of the robot with a tablet and a patient with a hand disorder.

The tablet application processes real-time wrist-extension values and uses them to guide the patient’s movements through an animated cat avatar. When the hand nears the top position, the avatar prompts the user to lower it; conversely, when the hand is down, it instructs extension. If the user does not respond, the command repeats cyclically until planned rest (Figure 8).

**Figure 9:**
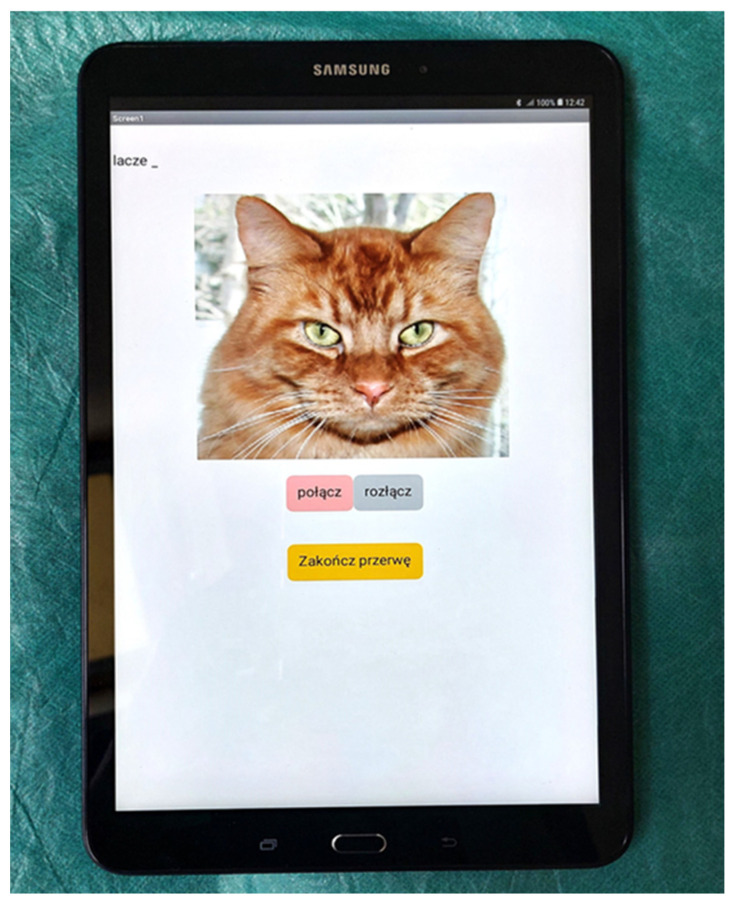
Tablet with audiovisual application (non-English: Alternatively: visible: cat avatar, connect/disconnect buttons, go to break).

The patient and therapist followed the following steps during the sessions:

1. Device configuration (Figure 9). The device is switched on with a button. The paretic hand is placed in the larger, balloon-based unit (the “follower”), while the healthy hand is placed in the smaller, flat unit (the “leader”). The patient lifts the healthy hand to set the range of motion for wrist extension, which the device records using a button-based interface. These settings are then translated for the affected hand. Exercises can be performed in either a sitting or lying position. (Note that at the time of this study, the force-estimation safety feature, described in the previous section, was not yet utilized.)
2. Application setup. The tablet application is activated and connects to the device via Bluetooth. A cat avatar delivers voice prompts—such as “Please extend the hand”, “Hand up”, or “Now down”—based on 10 s intervals and the user’s current hand position.
3. Timed exercise and rest. After 10 min of continuous training, the application switches to a 5 min rest period, represented by a “sleeping” cat on-screen.
4. Repetition. These exercise-and-rest cycles are repeated three times to complete the session.

**Figure 10:**
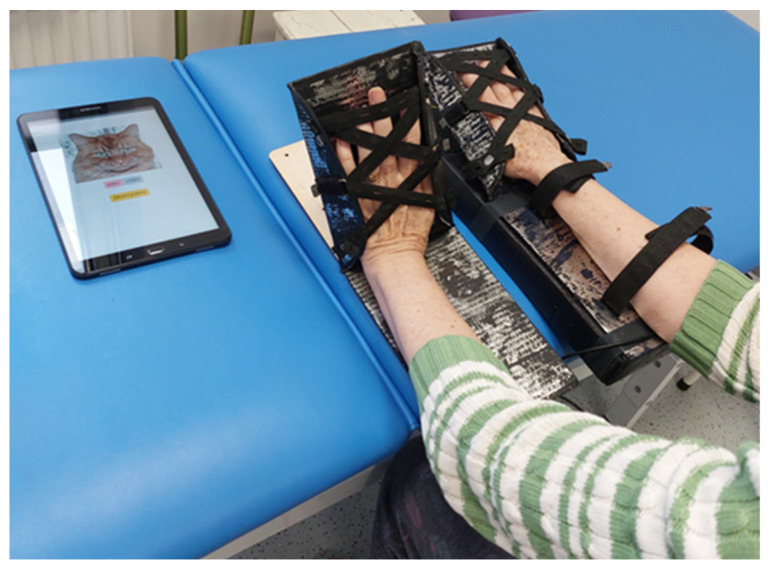
Patient therapy session in sitting position.

The study was performed in the sitting and lying positions (Figure 10).

Throughout the session, the device provides ongoing feedback on hand position. The cat avatar reinforces instructions by rephrasing commands if the user does not respond, mirroring a real-world therapist’s approach and sustaining patient engagement. This design aims to help patients feel supported and safe, encouraging consistent participation. We hypothesize that a straightforward wrist-extension device, paired with an audiovisual application that offers step-by-step guidance, can significantly enhance both motor and functional recovery for post-stroke patients.

### 2.3. Study Design

**Figure 11:**
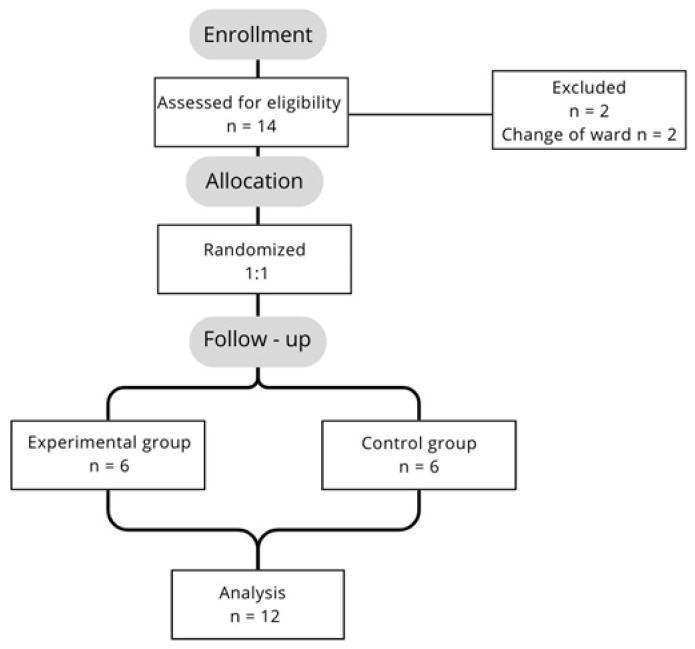
Schematic of the survey structure.

A total of 14 ischemic stroke patients were recruited. During the second week of the study, 2 patients were transferred to another ward due to progression of post-stroke medical complications and were excluded from the analysis. In total, 12 patients completed the entire trial, and their results were analyzed. Patients received printed information about the study design and signed an informed consent form to participate in the study. The experimental and control groups were randomized in a 1:1 ratio (Figure 11). The study was conducted in accordance with the tenets of the Declaration of Helsinki. The study was conducted at the Department of Neurological Rehabilitation at the Karol Jonscher Municipal Medical Center in Poland. We received approval from the head of the department to conduct the study. The study is part of a project funded by the National Center for Research and Development of Poland, entitled “Single-use and personalizable devices for hand rehabilitation” (0203/L-11/2019).

Patients were recruited into the study according to the inclusion and exclusion criteria:

Inclusion criteria for the study: patients diagnosed with stroke, MMSE (Mini-Mental State Examination) score of more than 24—not indicating significant cognitive impairment, preventing comprehension of instructions and participation in the study. Exclusion criteria for the study: inflammatory diseases with fever, severe general condition, systemic diseases, severe cardiovascular diseases (post myocardial infarction status of less than 30 days, unstable angina pectoris, cardiac failure, severe arrhythmia), impaired command comprehension and perceptual impairment or general significant deterioration, uncompensated metabolic and endocrine diseases, respiratory failure.

The age of the study participants ranged from 47 to 84 years. The characteristics of the patients are shown in the table below (Table 1).

Patients performed the exercises for 4 weeks, during which 20 treatment sessions were successfully completed. A training session lasted 45 min and included three sets of 10 min exercises and a 5 min break. The device was designed so that the weak hand mimicked the healthy hand, similarly to mirror therapy. The exercises performed consisted of stretching the wrist joint without causing pain. The control group performed similar exercises. All patients in the study received routine neurological rehabilitation in the department six times a week, excluding Sundays. Patients received psychological, speech, and occupational therapy in addition to conventional rehabilitation.

We performed 20 therapy sessions for 4 weeks, to examine recovery, return of upper-limb function, and the patient condition; we used the Fugl–Mayer Assessment for the Upper Extremity (FMA-UE) and modified Rankin Scale (mRS). To verify the cognitive improvement, we used the Montreal Cognitive Assessment (MoCA) and Mini-Mental State Examination (MMSE). The patient’s degree of independence was assessed using the Barthel Index (BI). We used the Portable Spasticity Assessment Device (PSAD, Denmark) to objectively measure the range of motion of the wrist joint extension and flexion. The patient was tested 5 times: once before the start of the study (1 trial) and always at the end of the week in which the rehabilitation and examination was carried out (4 trials every Friday).

## 3. Results

**Figure 12:**
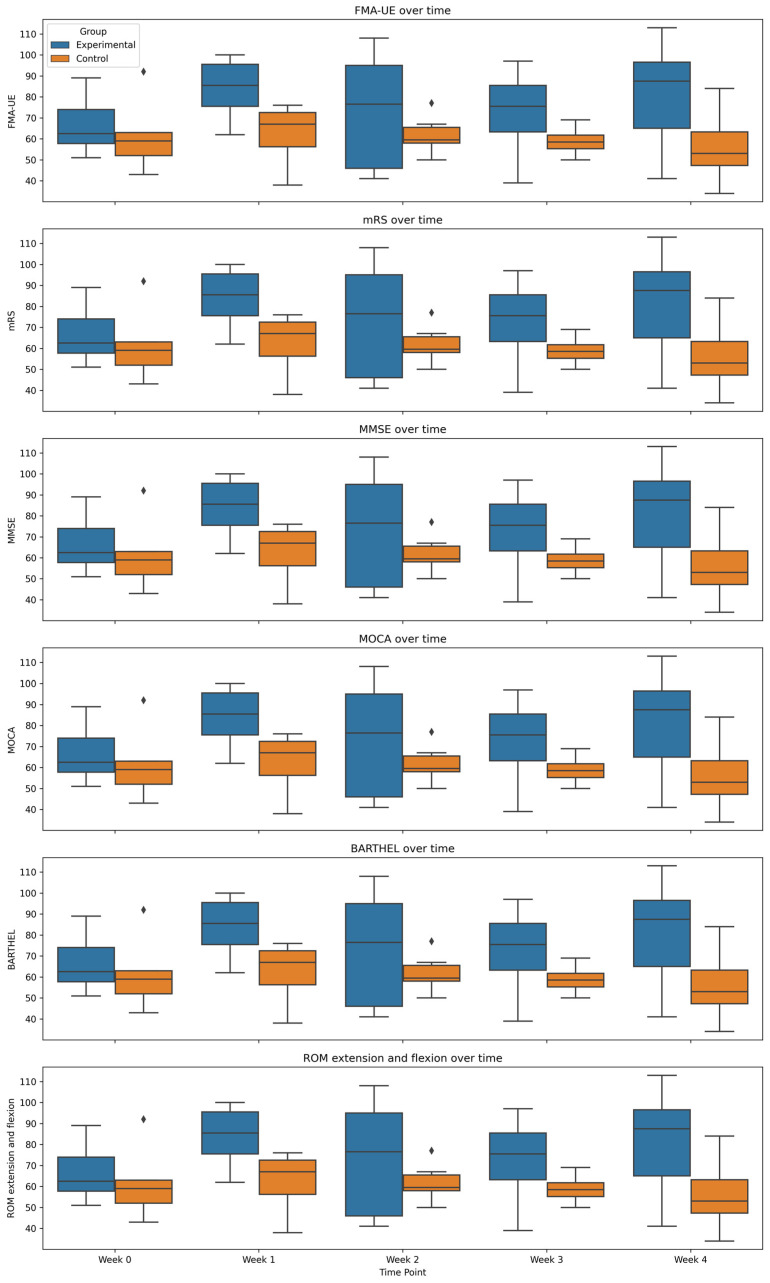
Box-plot of results obtained for experimental and control groups in time. X axis—weeks of study.

Statistical analyses were conducted separately for the experimental and control groups. All computations were performed using Statsmodels 0.13.5and SciPy 1.7.3. First, we applied an independent-samples t-test to assess mean changes from baseline (Week 0) to the final assessment (Week 4) for each clinical measure—FMA-UE, mRS, MMSE, MOCA, BI, and ROM. A maximum of 126 points can be obtained on the FMA-EU scale. The authors considered seven subsections: A/B/C/D/H/I/J. Subsections A–D relate to motor function for 66 points. Then, H-I-J cover sensation, passive joint movement, and joint pain for 60 points. A comparison of changes in patients from week 0 to week 4 is shown in Figure 12.

Overall, the experimental group showed higher scores on FMA-UE, mRS, MMSE, MOCA, BI, and ROM compared to the control group, with the most pronounced improvements seen in the last week of the study. The control group demonstrated smaller changes in these measures. Notably, the Barthel Index (BI) was the only measure that reached statistical significance using the *t*-test (*p* = 0.02), suggesting that the experimental device significantly enhanced functional independence and self-care. Patients in the experimental group scored, on average, 12.5 points higher than the control group in the final week. Effect sizes (Cohen’s d) were large or moderate for most outcomes (MMSE, MOCA, Barthel, and ROM), indicating clinically meaningful improvements, though not all passed the significance threshold—likely due to the small sample size. Table 2 summarizes the statistical comparisons of mean changes in outcome measures before and after the 4-week intervention.

The FMA-UE demonstrated a large effect size (Cohen’s d = 1.18). Although not statistically significant (*p* = 0.07), such a large effect size suggests meaningful clinical improvement. In fact, the experimental group showed an 18.83-point average increase on FMA-UE, compared to only 4.17 points in the control group. Wrist range of motion improved by 14.5° in the experimental group but worsened by 5.17° in the control group.

The scores obtained for the mRS scale should be interpreted differently from those of the other scales. In this case, the lower the score obtained for the mRS scale, the better the score for the patient, indicating less disability. The average change in mRS scores for both groups is small and negative, indicating a slight improvement in both groups. In most cases, it is an improvement of one level on this 6-level scale. Cohen’s d coefficient (d = −0.3131) shows a small effect size, indicating a small impact.

Because of the small sample size and the potential for non-normal distributions, a Mann–Whitney U test was performed as a secondary, non-parametric analysis. This approach provides additional confirmation when parametric assumptions may not be fully met. Table 3 displays the Mann–Whitney results, alongside Cliff’s delta (δ), which measures the magnitude of differences between groups.

Statistically significant differences (*p* < 0.05) again emerged for the FMA-UE and Barthel Index (BI), confirming that the experimental group’s improvement was unlikely due to chance. The largest Cliff’s delta (>0.8) also occurred for the BI, reflecting a very large effect. Large but non-significant differences were noted for ROM, ADL, and MMSE, aligning with the *t*-test results but falling short of statistical significance. Importantly, the mRS (scored inversely, where lower is better) showed only a minor average improvement in both groups, with a corresponding small effect size (Cohen’s d = −0.31).

## 4. Discussion

Stroke is a serious cerebrovascular disease that significantly compromises motor function in the limbs. During inpatient rehabilitation, the patients in our study showed notable improvements in neurological status. A common challenge for post-stroke patients is uncontrolled flexion synergy in the hemiparetic limb, which particularly impedes control of the wrist extensors [44,45]. To address this, we developed robotic exercises specifically targeting wrist extension—an area patients often find difficult to manage. Before admission to the study, all participants underwent physical rehabilitation and were evaluated by a medical rehabilitation specialist, who determined that they were suitable for further rehabilitation in a neurological rehabilitation unit. Following therapy, patients had reduced disability as measured by the mRS, with the experimental group demonstrating more pronounced gains. Improvements were also observed in cognitive function, muscle activation, wrist range of motion (assessed via PSAD, Denmark), overall upper-limb recovery, and independence.

Robotic rehabilitation is gaining popularity in stroke therapy due to its ability to perform repetitive, task-specific exercises, which can promote neuroplasticity [46]. Research suggests that 400–600 repetitions of functional upper limb exercises per day may be required to induce meaningful cortical reorganization [47]. In our pilot study, 12 stroke patients trained with the Balonikotron on weekdays for four weeks, totaling 900 min (15 h) of additional therapy. Providing the same intensity of training through hospital staff alone would be impractical in a public healthcare setting, given resource constraints [48]. Notably, participants in the experimental group required assistance only for tablet activation, as both hands were occupied—one with the balloon device (follower) and the other with the flat device (leader).

There was a significant improvement in ADL independence. There was an upward trend in cognitive function, range of motion, and upper-limb recovery. A significant improvement emerged in ADL independence, with notable gains in the Barthel Index of self-care and functional independence. Patients in the experimental group scored an average of 12.5 points higher on the Barthel Index in the last week of therapy compared to those receiving only conventional inpatient rehabilitation. This finding aligns with the work of Singh et al. (2019), who reported that an exoskeleton device improved FMA-UE and Barthel scores in subacute and chronic stroke patients [49]. Although our FMA-UE results did not reach statistical significance using the independent-samples *t*-test, the experimental group showed an 18.83-point mean improvement, indicating the potential efficacy of robot-assisted wrist rehabilitation as a safe and effective additional therapy. This conclusion is supported by Mazzoleni et al. (2018), who also noted significant FMA-UE gains in patients using a wrist-robot device [50].

**Figure 13:**
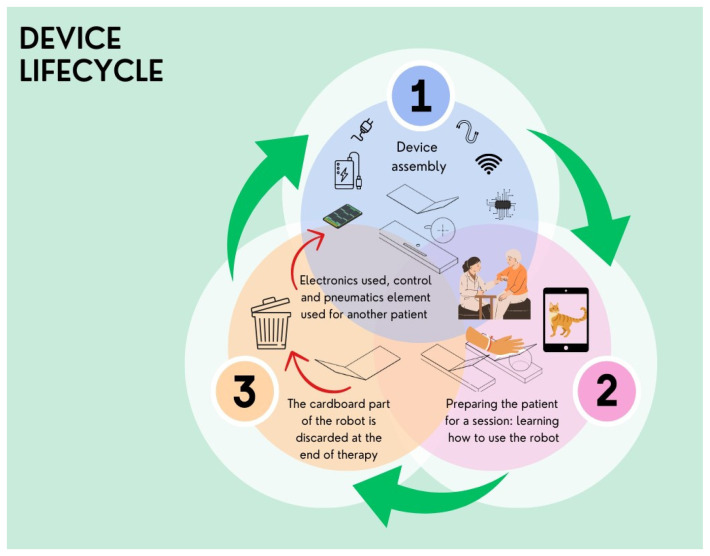
Device lifecycle, in which the top cardboard element that contacts with the patient’s skin is exchanged.

Evaluating the Balonikotron in its current form revealed both benefits and challenges. Because the device occupies both hands, continuous supervision is often necessary. Over four weeks of use in a hospital setting, the device functioned without malfunction, maintaining the integrity of its cardboard components and showing no signs of cable damage. These observations suggest that the Balonikotron could transition into a home setting, although family members might need to assist. By contrast, in a hospital environment—where stringent disinfection protocols are mandatory—its single-patient, disposable cardboard design helps minimize infection risk. Because of its low-cost design and compact size, the device could feasibly be integrated into ongoing rehabilitation outside the hospital. Li et al. (2021) emphasize that successful home-based rehabilitation devices must address usability, software, safety, and functionality within ADLs. Our robot facilitates wrist extension, which is essential for grasping and manipulating objects. Moreover, its software requirements are minimal, functioning on most Android devices. We encountered no safety issues during the study. While cost and spatial constraints are potential obstacles, using cardboard in the robot’s construction and limiting the mechanism to a single balloon kept the design both affordable and space-saving. The cost of the robot and the space it occupies are listed in the barriers to home implementation [51]. Beyond its cost advantages, the robot is single-patient use, portable, and easily set up, allowing for usage at home, in the hospital room, or in a dedicated exercise area. It also offers an environmentally conscious approach: after a training cycle, the cardboard housing and balloon are discarded, with the internal electronics reused. This cyclical design avoids cross-contamination and supports sustainable usage (Figure 13).

The device has features setting it apart from other rehabilitation robots:

### 4.1. Single-Patient Use

This device is intended for use by a single patient at a time. Its cardboard structure allows swift replacement when switching users, minimizing cross-contamination risks. Because each unit is dedicated to one patient, less frequent cleaning is needed, further enhancing hygiene.

### 4.2. Portable

Weighing only about 1 kg, the Balonikotron is easily transported or lifted by a single healthy hand. Its compact size enables use in multiple settings—such as a patient’s bed, desk, or garden—rather than restricting training to a clinical facility. The tablet that runs the application also has standard dimensions, making it simple to place on various surfaces without consuming excessive space. This portability extends access to every patient in a hospital ward.

### 4.3. Wide Availability

Most components (e.g., valve, pneumatic pump) are off-the-shelf and affordable, and the remaining parts (cardboard framework, custom electronics) rely on low-cost manufacturing processes (e.g., cutting, creasing with a plotter). Additionally, the application can run on any standard tablet, further reducing barriers to adoption. Together, these factors aim to bring affordable robotic rehabilitation to both hospital patients and individuals pursuing home-based recovery.

The Balonikotron employs an eco-conscious lifecycle. First, the device is assembled using cardboard, electronics, and a balloon. Next, patients receive training and guidance on its proper use. Finally, once the rehabilitation sessions conclude, the cardboard housing and balloon are disposed of—preventing cross-contamination and limiting the spread of pathogenic microorganisms—while the core electronics can be reused for new patients. This cyclical design conserves resources, ensures hygiene, and offers an environmentally responsible approach to repeated device usage.

Daily conventional rehabilitation in the hospital can often be monotonous for patients [52]. Robot-assisted rehabilitation, on the other hand, can boost motivation and engagement by incorporating virtual games and interactive applications [53]. Effective neurological training requires numerous repetitions and task-specific movements, which pose significant challenges for healthcare systems grappling with staffing constraints, high patient loads, and other limited resources [37,54]. Robot-assisted rehabilitation can relieve some of the pressure on medical teams while providing patients with consistent, repetitive training. Furthermore, robots may be effectively implemented for home-based rehabilitation in the future [51]. In our study, no patient withdrew because of a lack of confidence in the proposed robotic rehabilitation; in fact, participants readily embraced this modern, complementary approach.

During the 4-week rehabilitation program involving the Balonikotron device and an audiovisual application, patients provided feedback on potential improvements. For instance, they felt that the 5-min rest breaks were too long. In the first week, these intervals were not problematic because patients were adapting to the therapy and experiencing increased hand pain. However, by the final week, many reported that they no longer needed such extended pauses and preferred to continue exercising. This was an unforeseen outcome not anticipated by the research team.

Additionally, halfway through the study, patients expressed a desire for greater variety in the animal characters within the application. Although some appreciated the cat—which elicited positive emotions—others wished to see cats with different coat colors or entirely different animals. Interestingly, the red cat became a frequent topic of conversation among patients in the ward, sparking curiosity about the device’s functionality and the progress of fellow participants.

Hand swelling and edema are common concerns for post-stroke patients during rehabilitation [55]. However, throughout the entire training program, no patient experienced swelling, redness, or localized pruritic lesions related to ongoing wrist arthritis, suggesting that the therapy exerts a gentle effect on the affected hand.

Exercise can improve both motor and cognitive function in healthy older adults as well as those with neurological conditions [56,57]. Yao et al. (2022) explored Baduanjin imagery and exercise, comparing the two forms of training and noting positive effects on cognitive function. Baduanjin imagery, incorporating video viewing and mental practice, may help mitigate cognitive decline associated with aging [58]. In our study, in addition to standard neurological rehabilitation—psychological, speech, and occupational therapy—patients trained for four weeks using the Balonikotron wrist-extension device. Alongside two electronic platforms, the device included a tablet application featuring a cat avatar. This cat served as both a companion and a guide, instructing the patient when to perform a wrist extension, return to the starting position, or take a break. Because the tablet was positioned behind the device, the patient’s attention remained on the affected hand, which was mimicking the movement of the healthy hand, and on the application that directed each exercise.

In recent years, there has been a growing body of literature showing positive effects on cognitive function [36]. The exact mechanism telling us how physical training positively affects cognitive function has still not been explained. Therefore, it is not possible to unequivocally answer that exercise is the main driver of improvement [59]. The repetitive wrist-extension exercise provided by the device supports the mechanism of neuroplasticity in mirror therapy. Neuronal reorganization and adaptation are enabled by the features of repetition and the acquisition of patient experience [60]. A 2018 Cochrane review confirms the efficacy of MT in reducing limb pain, decreasing impairment, and improving motor function in performing activities of daily living in stroke patients [20].

Other studies report that combining conventional upper-limb rehabilitation with mirror therapy yields substantial benefits, including improvements in cognitive function rehabilitation [36,61,62]. Consequently, we designed the Balonikotron with a dual mechanism so that the paretic hand could mimic the healthy hand, aligning with mirror therapy principles. After two weeks, patients who engaged in robotic training showed notable improvements in both upper-limb function and cognitive ability. Although those who practiced only wrist-extension exercises with standard instructions also improved, their progress was slower. Gandhi et al. (2020) found that mirror therapy can benefit post-stroke patients at any phase, despite not specifically examining cognitive outcomes [28]. The illusion of healthy-hand movement can stimulate the premotor cortex through visual pathways [62], and “mirror neurons” are activated when the brain acquires new motor skills by observing and visualizing movements [61]. Further research is needed to fully understand how these processes affect cognition.

Stroke patients often experience spontaneous recovery within weeks or months of the initial event. Factors such as stroke size, location, patient age, and pre-existing conditions all influence the rate of improvement. Spontaneous recovery is most pronounced in the first three months [63], yet neuroplasticity allows for brain regeneration even in the chronic phase [64]. Thus, physical exercise combined with visual engagement may help improve cognitive function for post-stroke individuals. In our study, the repeated wrist-extension exercises, coupled with app-based visual feedback, complemented the existing psychological therapy patients received in the ward. 

Throughout the study, no adverse incidents occurred—such as balloon bursts, unexpected interruptions, or device shutdowns. Patients were able to independently attach the devices (leader and follower) to both their impaired and healthy hands, ensuring safe and effective rehabilitation.

There are a few limitations to our study. As this was a pilot investigation involving 12 ischemic stroke patients, future research with larger sample sizes is needed to confirm and strengthen our findings. Measurements obtained using the PSAD may appear inflated for wrist extension because they were taken from a neutral (partially flexed) position to the maximum extension. Due to participants’ low muscle strength and significant paresis, the neutral position was already relatively flexed, which artificially increased the measured extension. In subsequent studies, we plan to evaluate flexion and extension parameters separately.

The device itself has certain constraints. During operation, the balloon’s pneumatic inflation system produces noise, which could theoretically disturb some patients, although none in our study reported it as problematic. Additionally, the cardboard housing—while cost-effective and easy to replace—may not align with patients’ expectations of medical equipment typically made of plastic, raising concerns about durability.

Another consideration is that the robot targets a specific patient population. Post-stroke patients with high spasticity may struggle to align the wrist or unclench the fist enough to use the device effectively. In our study, several participants scored a 2 on the Modified Ashworth Scale (MAS), yet encountered no difficulties, suggesting that the Balonikotron may be suitable for those with MAS scores ≤ 2, as long as their spasticity does not worsen. Due to its mirror-therapy-like design, the device is also appropriate for patients with sensory disorders, including profound sensory impairments. However, the Balonikotron only partially replicates classic mirror therapy: the dual-platform approach was intended to test mirror-therapy principles but does not identically reproduce them.

## 5. Conclusions

Controlled wrist-extension exercises with elements of mirror therapy can lead to improved hand function, including self-care and independence. The results of our pilot study suggest that the Balonikotron device, which uses the principles of mirror therapy, may serve as a valuable adjunct to conventional rehabilitation for post-stroke patients with hemiparetic hands. Our study demonstrates that a relatively simple device like the Balonikotron, combined with an audiovisual cat application, is effective in aiding hand rehabilitation after stroke. The device offers a promising and accessible approach to post-stroke recovery by integrating engaging and motivating elements, ultimately supporting both physical and cognitive rehabilitation.

## 6. Patents

Igor Zubrycki is one of the authors of Polish patent Pat.243910 “Pneumatic device for training passive extension in the wrist joint”, which describes the basic invention of the Balonikotron device.

## Figures and Tables

**Table 1:** Patient characteristics (n = 12).

n = 12	Gender	Age	Time Since Stroke [months]	Side of Paresis
1	M	69	<1	R
2	M	52	<1	R
3	M	73	1	R
4	M	84	1	R
5	W	60	<1	R
6	M	72	2	L
7	M	70	22	L
8	W	66	2	R
9	M	47	60	L
10	M	72	1	L
11	W	78	1	L
12	M	66	19	L
	25% W 75% M	67.4 ± 10.4	9.3 ± 17.7	50% L 50% R

<1~0.75. Arithmetic mean ± SD.

**Table 2:** Details of the statistical results obtained for the experimental and control groups.

	Experimental Group n = 6	Control Group n = 6
Independent-samples *t*-test results for Barthel Index		
Mean Change	19.17	6.67
SD	9.17	5.16
t-statistic	2.90	
*p*-value	0.02	
Cohen’s d	1.68	
Independent-samples *t*-test results for MMSE		
Mean Change	1.17	0.33
SD	1.17	0.82
t-statistic	1.43	
*p*-value	0.19	
Cohen’s d	0.82	
Independent-samples *t*-test results for mRS		
Mean Change	−0.50	−0.33
SD	0.55	0.52
t-statistic	−0.54	
*p*-value	0.6	
Cohen’s d	−0.31	
Independent-samples *t*-test results for FMA-UE		
Mean Change	18.83	4.17
SD	15.12	8.91
t-statistic	2.05	
*p*-value	0.07	
Cohen’s d	1.18	
Independent-samples *t*-test results for MOCA		
Mean Change	5.17	3.50
SD	2.14	1.05
t-statistic	0.72	
*p*-value	0.13	
Cohen’s d	0.99	
Independent-samples *t*-test results for ROM		
Mean Change	14.50	−5.17
SD	23.94	20.38
t-statistic	1.53	
*p*-value	0.16	
Cohen’s d	0.88	

**Table 3:** Mann–Whitney U Test details.

Clinical Scale	Mann–Whitney U	*p*-Value *	Cliff’s Delta δ
FMA-UE	31.0	0.045	0.722 (large)
mRS	15.0	0.640	−0.167 (small)
MMSE	26.5	0.151	0.472 (medium)
MOCA	26.5	0.187	0.472 (medium)
BI	34.0	0.009	0.889 (large)
ROM	28.5	0.109	0.583 (large)

* statistical significance at *p* < 0.05.

## Data Availability

Data are contained within the article.

## Abbreviations

FMA-UE	The Fugl-Meyer Assessment for Upper Extremity
mRS	Modified Rankin Scale
MMSE	The Mini-Mental State Examination
MOCA	The Montreal Cognitive Assessment
ROM	Range of Motion
BI	Barthel Index
PSAD	Portable Spasticity Assessment Device
EG	Experimental Group
CG	Control Group
